# Defining the value of CD56, CK19, Galectin 3 and HBME-1 in diagnosis of follicular cell derived lesions of thyroid with systematic review of literature

**DOI:** 10.1186/s13000-015-0428-4

**Published:** 2015-10-26

**Authors:** Duško Dunđerović, Jasmina Marković Lipkovski, Ivan Boričic, Ivan Soldatović, Vesna Božic, Dubravka Cvejić, Svetislav Tatić

**Affiliations:** Institute of Pathology, Faculty of Medicine, University of Belgrade, dr Subotica 1, 11000 Belgrade, Serbia; Department of Pathology, Centre for Endocrine Surgery, Clinic for Diabetes and Metabolic Diseases, Clinical Center of Serbia, Dr. Koste Todorovića 8, 11000 Belgrade, Serbia; Institute for the application of nuclear energy, Belgrade, Serbia, Banatska 31b, Zemun, 11080 Zemun, Serbia; Faculty of Medicine, University of Belgrade, dr Subotica 8, 11000 Belgrade, Serbia

**Keywords:** Thyroid, Immunohistochemistry, Tissue Microarray, CD56, CK19, Galectin 3, HBME-1

## Abstract

**Background:**

Nodular follicular lesions of thyroid gland comprise benign and malignant neoplasms, as well as some forms of hyperplasia. “Follicular” refers to origin of cells and in the same time to growth pattern - building follicles. Nodular follicular thyroid lesions have in common many morphological features, therefore attempts were made to define additional criteria for distinction between follicular adenoma, follicular carcinoma and follicular variant of papillary carcinoma. Increasing number of immunohistochemical markers is in the continual process of evaluation.

**Methods:**

Tissue microarrays incorporating, total 201 cases, out of which 122 malignant and 79 benign follicular lesions, including neoplastic and non-neoplastic, were constructed and immunostained with antibodies to CD56, CK19, Galectin-3, HBME-1. Tissue cores were exclusively being acquired from tumour/lesion on interface with normal thyroid tissue. A systematic review of literature was done for period from the year 2001 to present time.

**Results:**

All analysed markers may make a difference between benign lesions/tumours from differentiated thyroid carcinomas (*p* = <0.01, for all markers). Expression of all markers is significantly higher in papillary carcinoma than in follicular adenoma (*p* < 0.01). Statistically significant difference in expression of Galectin-3 and CD56 between follicular carcinoma and follicular adenoma was registered (*p* = 0.043; *p* = 0.028, respectively). The only marker which expression showed statistically significant difference between adenoma and carcinoma of Hurthle cells was Galectin 3 (*p* = 0.041). CK19 and HBME-1 were significantly expressed more in papillary carcinoma as compared to follicular carcinoma.

**Conclusion:**

Galectin 3 is most sensitive marker for malignancy, while loss of expression of CD56 is very specific for malignancy. Expected co-expression for combination of markers in diagnosis of follicular lesions decreases sensitivity and increases specificity for malignancy.

## Background

Pathology of thyroid gland is diverse. Nevertheless, from practical reasons, all lesions can be divided into two groups, with nodular and diffuse pattern of growth. The former group clinically manifested as thyroid nodules, comprise benign and malignant neoplasms, as well as some forms of hyperplasia [1].

Nodules of thyroid gland are very frequent. It was estimated that 5 % of general population develop clinically obvious nodule. With introduction of better ultrasound facilities, the detection of non palpable nodules is on the rise, 20–67 % of detected non palpable thyroid nodules [2].

Term “follicular” in thyroid gland has dual connotation, to have origin from follicular cells or building follicles (designating follicular pattern of growth). Lesions with follicular growth pattern could be further classified relative to size of follicles (micro-, macrofollicular), or in respect to presence of capsule (totally /partially encapsulated, non encapsulated). Universally, majority of follicular lesions could be classified into benign and malignant category. According to presence or absence of features in parenthesis (capsule, vascular/capsular invasion, papillary carcinoma type nuclei) we classify them as: adenomatoid nodules and adenomas, papillary thyroid carcinoma (PTC), follicular thyroid carcinoma (FTC), well differentiated tumours of uncertain malignant potential (WDT-UMP), follicular tumour of uncertain malignant potential (FT-UMP), well differentiated carcinoma, NOS, Hurthle cell adenoma/carcinoma [3–7]. Follicular nodular thyroid lesions have in common many morphological features, which frankly put a burden on pathologist while trying to make diagnosis on H&E slides. Even amongst experienced endocrine pathologist there exists inter-observer variability. Furthermore, intra-observer variability is seen when they review the same H&E slides after some period of time [8].

Attempts were made to define additional criteria for distinction of follicular adenoma (FA) from follicular carcinoma and follicular variant of papillary carcinoma, and between two later mentioned. Increasing number of immunohistochemical markers are being tested, and some are promising like CD56, Hector Battifora Mesothelial 1 (HBME-1), Galectin 3 (Gal-3) and Cytokeratin 19 (CK19) considering differential diagnosis, nevertheless, none of them is individually conclusive [6, 9].

The aim of this study was to test sufficient number of different follicular thyroid lesions using for that purpose tissue microarray (TMA) technology, exploiting all four above mentioned markers. Our intention is to try to obtain answers to following questions: Can they distinct benign from malignant lesions?; Can they differentiate between papillary carcinoma (especially follicular variant) from follicular carcinoma or adenoma; Could they differentiate follicular adenoma from follicular carcinoma? We hypothesized that not just one combination but acceptable number of well-tailored combinations of immunohistochemical markers should suit for different differential diagnostic combinations. Elaborated review of literature on expression of CD56, CK19, HBME-1, Gal-3 is also provided.

## Methods

### Case selection

This retrospective study was conducted on 201 cases of thyroid lesions, including 44 males and 157 females. Majority of cases were from 2013^th^, and 2014^th^, but due to the paucity of cases with follicular thyroid carcinoma, aforementioned cases were selected retrospectively all the way till the year of 2007^th^. The research was approved by Ethical committee of Medical Faculty, University in Belgrade. Cases were selected from archives of Department for endocrine pathology, Center for endocrine surgery, Clinical centre of Serbia. Glass slides (on average 7 per case) were retrieved and evaluated by three experienced endocrine pathologists, who were unaware of clinical information and previous diagnosis. Diagnosis for problematic cases was made by consensus of two pathologists. Examination comprised 122 malignant and 79 benign follicular lesions. Only tumours with diameter larger than 5 mm were included in the study.

### TMA

Four high density TMAs were constructed manually. Area of interest was the zone right beneath tumour capsule or just on invasive tumours front. Previously marked area of interest on slides was translated to corresponding regions of donor paraffin blocks. Needle with inner diameter of 1.1 mm was used to create and transfer tissue cores (0.785 mm^2^ cross cut surface area) in recipient paraffin blocks. Two cores were taken from every lesion. Cases with at least one section across all slides were regarded as valid. Tissue cores with external controls were included in all TMAs. Final TMA blocks consisted of 104 cores (13x8), plus five control tissue cores. Control tissues included in TMA were normal thyroid tissue, follicular thyroid adenoma, mucosa of appendix (crypts positive to CK19 and Gal-3), serous membrane of appendix (mesothelial cell immunopositive for HBME-1), muscular layer of appendix (nerve fibers and ganglion cells positive for CD56).

### Immunohistochemistry

Immunohistohemical staining with CD56 (NOVOCASTRA, Clone 1B6, 1:50), HBME-1 (DAKO, Clone HBME-1, 1:50), CK19 (DAKO, Clone RCK 108, 1:50), Galectin-3 (R&D SYSTEMS, Clone 194804, 1:100) was done manually according to manufacturers instructions (Table 1).

**Table 1:** Immunohistochemistry

Antibody	Clone	Manufacturer	Dilution	Pretreatment	Incubation with primary antibody	Visualisation kit manufacturer	Visualisation kit
CD56 (NCAM)	1B6	Novocastra, UK	1:50	microwave oven, 20′, citrate buffer, pH6	1 h	Thermo Scientific™, USA	TL-125-HL
Cytokeratin 19	RCK108	Dako, Denmark a/s	1:100	enzime digestion, preteinase K, 10′, room temp	30′	Thermo Scientific™, USA	TL-125-HL
HBME-1	HBME-1	Dako,Denmark A/S	1:50	microwave oven, 20′, citrate buffer, pH6	1 h	Thermo Scientific™, USA	TL-125-HL
Human Galecin-3	194804	Techne/r&d systems, USA	1:100	microwave oven, 20′, citrate buffer, pH6	12 h, 4 °C	Thermo Scientific™, USA	TL-125-HL

### Evaluation of immunohistochemical staining

Cytoplasmatic ± nuclear immunoreactivity for Gal-3, membranous ± cytoplasmatic immunoreactivity for CK19, and membranous ± cytoplasmatic immunoreactivity for HBME-1 in more than 10 % of cells was considered as positive staining without regard to intensity of staining. In respect to distribution of staining we graded staining as 1, 2, 3, 4, when 11–25 %, 26–50 %, 51–75 %, 76–100 % of tumours cells show expression, respectively [10].

Membranous staining of follicular cells with CD56 was regarded as positive. On the grounds that CD56 expression is reduced or missing in thyroid carcinomas, positive result (or malignant profile) was scored as 1 when 0–10 % follicular cells is immunoreactive for CD56. Score 0 (negative) was in the case that equal or more than 11 % of follicular cells were positive for CD56 [11].

### Statistical analysis

Database and data analyses were done with IBM SPSS Statistics 20. Descriptive and analytical methods were used. For comparison of variables, parametric (*t* test, ANOVA) and non-parametric tests (chi square, Mann Whitney *U* test, Kruskal Wallis test) were employed. With objective to compare markers Receiver Operating Curve (ROC) was constructed. Sensitivity, specificity, positive and negative predictive values, and diagnostic odds ratio were calculated for single and combination of markers in program MedCalc® (Version 10.2.0.0). Probability values less than 0.05 were considered statistically significant.

### Review of literature

Review of literature was done in PUBMED for period from the year 2001 to present time. The following describers were used ((CK-19 and thyroid) OR (GALECTIN-3 and thyroid) OR (HBME-1 and thyroid) OR (CD56 and thyroid). We included studies which contain benign as well as malignant cases, and which employ tissue immunohistochemistry as technique. The following information was acquired: reference, number of benign and malignant cases, histological types of neoplasms studied and results (stratified into two groups: true positive, true negative, false positive, false negative). Our results were compared with different studies.

## Results

Average age of patients was 51 ± 14 years, with median of 53. Comparing benign with malignant group, matched relative to sex, and relative to age, we found no statistical difference between groups (*p* = .134; *p* = .051, respectively). The mean size of benign and malignant lesions was 3.3 cm (range, 0.5 to 9.0 cm) and 3.4 cm (range 0.5 to 12.0 cm), respectively (*p* = .719). Malignant group consisted of 87 papillary thyroid carcinomas, 15 follicular thyroid carcinomas and 20 Hurthle cell carcinomas. Benign lesions included 27 follicular adenomas, 10 Hurthle cell adenomas, 12 hyperplastic adenomas, and 30 nodular goiters (colloid adenomas). The group of papillary carcinomas was represented by 40 follicular type, 27 classic type, 9 mixed, 5 solid and 3 oxyphilic type carcinomas.

**Fig. 1:**
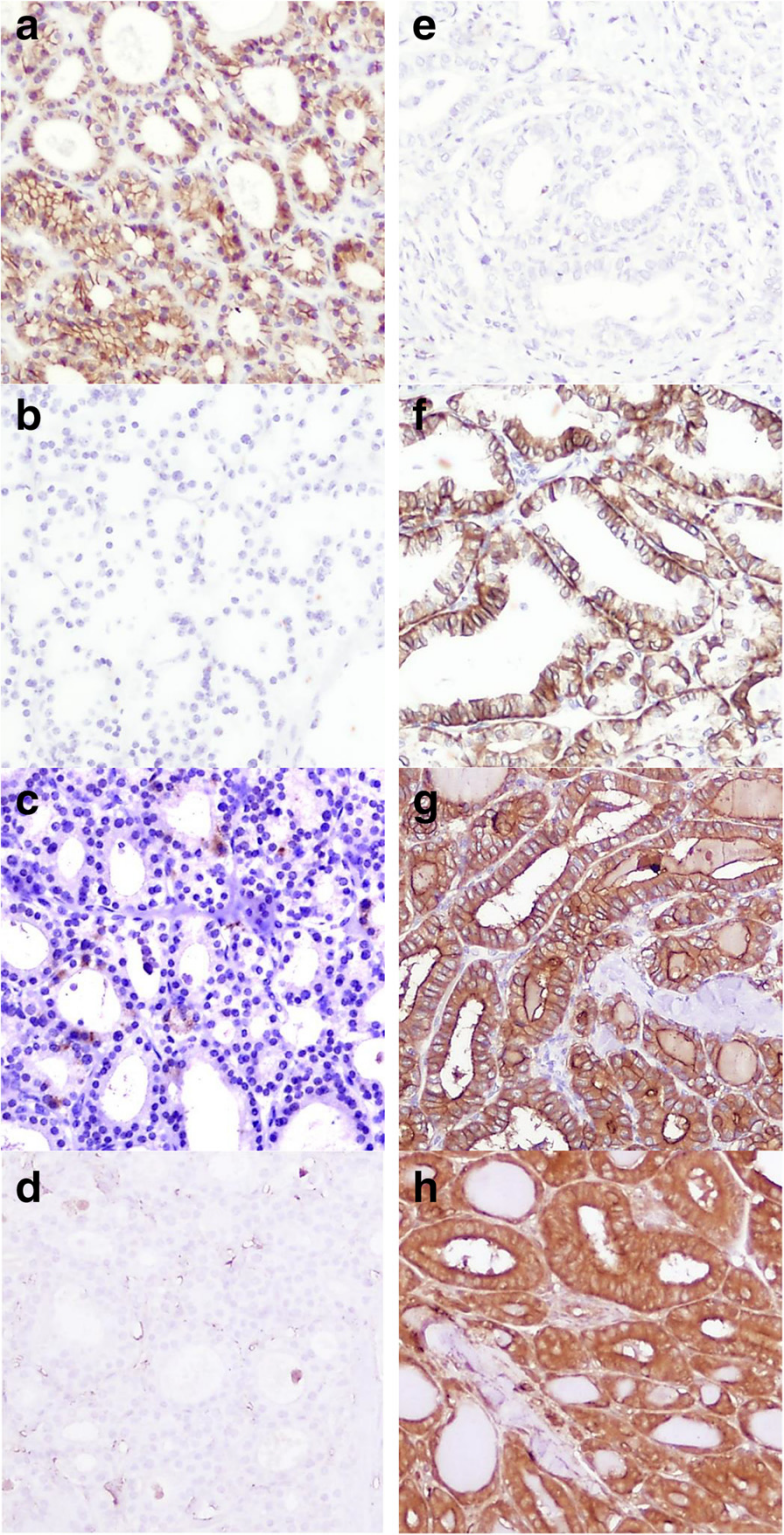
Expression of CD56, CK19, HBME-1, Galectin-3 in folicular adenoma and follicular variant of papillary carcinoma. **a** membranous immunoexpression of CD56 in follicular adenoma; (**b**): absence of immunoexpression of CK19 in follicular adenoma; (**c**): absence of immunoexpression of HBME-1 in follicular adenoma; (**d**): absence of imunoexpression of Galectin-3 in follicular adenoma; (**e**): loss of immunoexpression of CD56 in follicular variant of papillary thyroid carcinoma; (**f**): membranous and cytoplasmatic immunoexpression of CK 19 in follicular variant of papillary thyroid carcinoma; (**g**): membranous and cytoplasmatic immunoexpression of HBME-1 in follicular variant of papillary thyroid carcinoma; (**h**): cytoplasmatic immunoexpression of Galectin-3 in follicular variant of papillary thyroid carcinoma

**Fig. 2:**
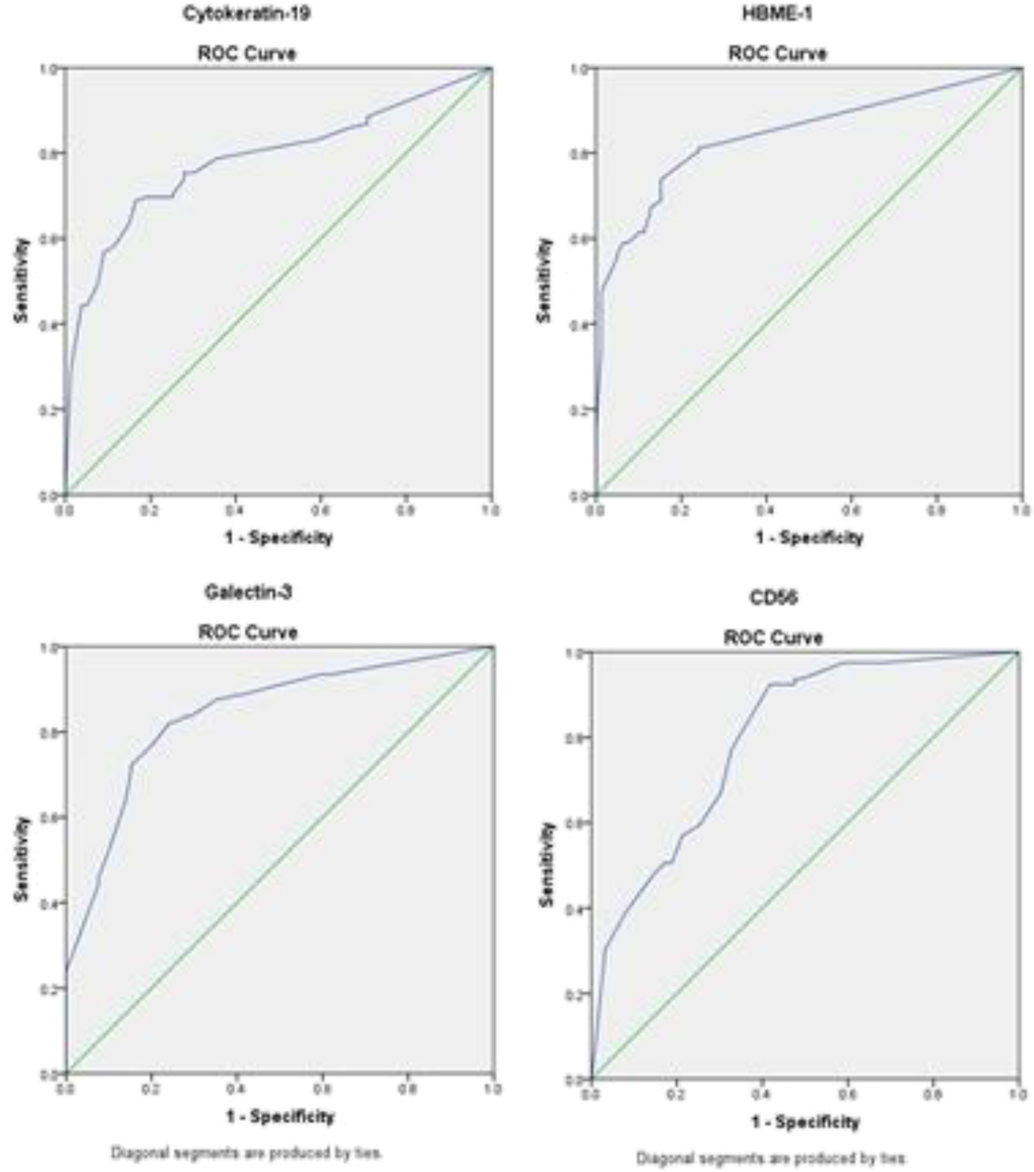
ROCs for CK19, HBME-1, Galectin-3, CD56. ROC Curve: receiver operating characteristic (ROC) curve

CK19 expression was present in 75 % of malignant tumours, generally with high intensity and wide distribution, and in 29.1 % of benign lesion, usually with weak and focal expression (*p* = .000) (Tables 2 and 3). Immunoreactivity was cytoplasmatic, sometimes with membranous accentuation (Fig. 1). Among carcinomas, difference in expression of CK19 existed between papillary carcinoma and follicular carcinoma (*p* = .000). Differences in expression were significant between papillary carcinoma and follicular adenoma (*p* = .000), as well as between follicular variant of papillary carcinoma and follicular carcinoma (*p* = .004) or adenoma (*p*=. 000). When we excluded papillary carcinoma from the sample we found statistical significance between malignant and benign group (*p* = 0.045). Significant difference in expression of CK19 was not found between follicular adenoma and follicular carcinoma (*p* = .433), or between Hurthle cell adenoma/carcinoma (*p* = .894). ROC curve (Fig. 2) was constructed, and value higher than 9.5 % of positive tumours cells had sensitivity of 75.4 %, and specificity 71 % for carcinoma (Table 4).

**Table 2:** Expression of CK19, HBME-1, Galectin-3, CD56 in benign and malignant tumors

Markers	Expression	Tumor			
		Benign		Malignant	
		N	%	N	%
CK 19	+	23	29.1 %	92	75.4 %
	-	56	70.9 %	30	24.6 %
HBME 1	+	12	15.2 %	87	71.3 %
	-	67	84.8 %	35	28.7 %
Galectin 3	+	28	35.4 %	108	88.5 %
	-	51	64.6 %	14	11.5 %
CD 56	+	6	7.6 %	71	58.2 %
	-	73	92.4 %	51	41.8 %

**Table 3:** Number and percent of cases with positive expression within different pathohistological entities

PH diagnosis	No of cases	CK 19		HBME 1		Galectin 3		CD 56^a^	
		N	%	N	%	N	%	N	%
Colloid adenoma	30	11	36.7 %	1	3.3 %	4	13.3 %	4	86.70 %
Follicular adenoma	27	6	22.2 %	4	14.8 %	11	40.7 %	1	96.30 %
Hurthle cell adenoma	10	6	60.0 %	7	70.0 %	5	50.0 %	1	90.00 %
Hyperplastic adenoma	12	0	0.0 %	0	0.0 %	8	66.7 %	0	100.00 %
Follicular carcinoma	15	5	33.3 %	4	26.7 %	11	73.3 %	4	73.30 %
Hurhle cell carcinoma	20	12	60.0 %	17	85.0 %	17	85.0 %	2	90.00 %
Papillary carcinoma	87	75	86.2 %	66	75.9 %	80	92.0 %	65	25.30 %

^a^immunopositivity in >10 % follicular cells

**Table 4:** Area under the curve

Test Result Variable(s):	Area	Std. Error^a^	Asymptotic Sig.^b^	Asymptotic 95 % Confidence Interval	
				Lower Bound	Upper Bound
CK19	.786	.032	.000	.723	.848
HBME-1	.842	.028	.000	.788	.896
Galectin-3	.841	.028	.000	.786	.897
CD56	.799	.031	.000	.739	.860

^a^Under the nonparametric assumption

^b^Null hypothesis: true area = 0.5

Malignant tumors expressed intensively and widely HBME-1 antigen than benign lesions (71.3 %; 15,2 %, respectively; *p* = .000). Variation in immunoreactivity was significant between papillary and follicular carcinoma (*p* = .000), papillary carcinoma and follicular adenoma (*p* = .000), and between follicular variant of papillary carcinoma and follicular carcinoma/adenoma (*p* = .009; *p* = .000 respectively). Once again, no significant difference was found comparing follicular adenoma/carcinoma (*p* = .465) in respect to HBME-1 expression. The values equal or higher than 7.5 % of follicular cells immunoreactive for HBME-1 had sensitivity of 71.3 % and specificity of 85 % for carcinoma.

Galectin-3 was expressed significantly more in malignant tumours (88.5 %) than in benign (35.4 %) (*p* = .000). Further, expression of Galectin-3 in papillary carcinoma and follicular variant of papillary carcinoma was notably higher than in follicular adenoma (*p* = .000; *p* = .001; respectively). We found no difference in expression between papillary and follicular carcinoma (*p* = 0.171), nor between follicular variant of papillary carcinoma and follicular carcinoma (*p* = 0.691). At last, follicular carcinoma had higher expression than follicular adenoma (0.043) and Hurthle cell carcinoma than Hurthle cell adenoma (*p* = 0.041). With cut off value of 7.5 % of follicular cells expressing Galectin-3, sensitivity is 89 %, and specificity is 65 % for carcinoma.

CD56 expression was lost in 58.2 % of malignant lesions and 7.6 % of benign lesions (*p* = .000). Papillary carcinoma lost expression in 74.7 % of cases, compared to follicular carcinomas 26.7 % (*p* = .006), and follicular adenoma 3.7 % (*p* = .000). No statistically significant difference in expression was observed between follicular variant of papillary carcinoma and follicular carcinoma (*p* = .282), but there is a difference between follicular variant of papillary carcinoma and follicular adenoma (*p* = .000). Interestingly, difference also existed between follicular carcinoma and follicular adenoma (*p* = 0.028), and between follicular carcinoma and benign thyroid lesions (without Hurthle cell adenomas; *p* = 0.006) Having insight in ROC coordinates, the values less than 12.5 % of follicular cells expressing CD56, have sensitivity of 58 % and specificity of 92.4 % for carcinoma.

Diagnostic value of all investigated markers and their combinations in differentiating malignant from benign thyroid follicular lesions was presented in a form of sensitivity, specificity, positive and negative likelihood ratios, disease prevalence, positive and negative predictive values, and odds ratio (Tables 5 and 6). Sensitivity and specificity for different combinations of markers in respect to various histopathological entities were presented in Table 7.

**Table 5:** Diagnostic value of tests in discrimination of malignant from benign thyroid lesions

For malignancy	HBME-1	Galectin-3	CK19	CD56
Sensitivity	71.31 %	88.52 %	75.41 %	58.20 %
Specificity	84.81 %	64.56 %	70.89 %	92.41 %
Positive Likelihood Ratio	4.69	2.5	2.59	7.66
Negative Likelihood Ratio	0.34	0.18	0.35	0.45
Disease prevalence	60.70 %	60.70 %	60.70 %	60.70 %
Positive Predictive Value	87.88 %	79.41 %	80.00 %	92.21 %
Negative Predictive Value	65.69 %	78.46 %	65.12 %	58.87 %
Odds ratio	13.8786	14.051	7.4667	16.9379

**Table 6:** Diagnostic value of markers combinations (all markers in combination positive) in discrimination of malignant from benign thyroid lesions

For malignancy	CD56&CK19	CD56&HBME1	CD56&Gal3	CK19&HBME1	CK19&Gal 3	HBME1&Gal 3	CD56&Gal 3&CK19	HBME-1&Gale3&CK19	CD56&Gal 3&HBME-1	CD56&CK19&HBME-1	CD56&CK19&HBME-1&Gal 3
Sensitivity	50.82 %	50.00 %	52.46 %	60.66 %	68.03 %	64.75 %	45.90 %	54.92 %	45.08 %	45.08 %	40.16 %
Specificity	94.94 %	98.73 %	97.47 %	89.87 %	88.61 %	92.41 %	97.47 %	93.67 %	98.73 %	98.73 %	98.73 %
Positive Likelihood Ratio	10.04	39.5	20.72	5.99	5.97	8.53	18.13	8.68	35.61	35.61	31.73
Negative Likelihood Ratio	0.52	0.51	0.49	0.44	0.36	0.38	0.56	0.48	0.56	0.56	0.61
Disease prevalence	60.70 %	60.70 %	60.70 %	60.70 %	60.70 %	60.70 %	60.70 %	60.70 %	60.70 %	60.70 %	60.70 %
Positive Predictive Value	93.94 %	98.39 %	96.97 %	90.24 %	90.22 %	92.94 %	96.55 %	93.06 %	98.21 %	98.21 %	98.00 %
Negative Predictive Value	55.56 %	56.12 %	57.04 %	59.66 %	64.22 %	62.93 %	53.85 %	57.36 %	53.79 %	53.79 %	51.66 %

**Table 7:** Sensitivity and specificity values of different markers combinations for different combinations of PH entities

All markers in combination were positive Sensitivity & Specificity for first in comparison		Malign Benign	PTC vs FA	FVPTC vs FA	FVPTC vs FTC	FTC vs FA	PTC vs non-tumor	HCC vs HCA
CD56_CK19	Sensitivity	50.8 %	65.5 %	50.0 %	50.0 %	20.0 %	65.5 %	10.0 %
	Specificity	94.9 %	100 %	100.0 %	85.7 %	100.0 %	92.9 %	90.0 %
CD56_HBME1	Sensitivity	50.0 %	65.5 %	55.0 %	55.0 %	13.3 %	65.5 %	10.0 %
	Specificity	98.7 %	100 %	100.0 %	92.9 %	100.0 %	100.0 %	90.0 %
CD56_GAL3	Sensitivity	52.5 %	69 %	57.5 %	57.5 %	20.0 %	69.0 %	5.0 %
	Specificity	97.5 %	100 %	100.0 %	85.7 %	100.0 %	97.6 %	90.0 %
HBME1_CK19	Sensitivity	60.7 %	67.8 %	52.5 %	52.5 %	20.0 %	67.8 %	60.0 %
	Specificity	89.9 %	92.6 %	92.6 %	85.7 %	92.6 %	97.6 %	50.0 %
GAL3_HBME1	Sensitivity	64.8 %	70.1 %	60.0 %	60.0 %	20.0 %	70.1 %	75.0 %
	Specificity	92.4 %	96.3 %	96.3 %	85.7 %	96.3 %	97.6 %	60.0 %
CK19_GAL3	Sensitivity	68.0 %	78.2 %	70.0 %	70.0 %	33.3 %	78.2 %	50.0 %
	Specificity	88.6 %	88.9 %	88.9 %	71.4 %	88.9 %	92.9 %	70.0 %
CD56_CK19_HBME1	Sensitivity	45.1 %	58.6 %	45.0 %	45.0 %	13.3 %	58.6 %	10.0 %
	Specificity	98.7 %	100 %	100.0 %	92.9 %	100.0 %	100.0 %	90.0 %
CD56_GAL3_HBME1	Sensitivity	45.1 %	59.8 %	50.0 %	50.0 %	13.3 %	59.8 %	5.0 %
	Specificity	98.7 %	100 %	100.0 %	92.9 %	100.0 %	100.0 %	90.0 %
CD56_GAL3_CK19	Sensitivity	45.9 %	59.8 %	45.0 %	45.0 %	20.0 %	59.8 %	5.0 %
	Specificity	97.5 %	100 %	100.0 %	85.7 %	100.0 %	97.6 %	90.0 %
HBME1_GAL3_CK19	Sensitivity	54.9 %	62.1 %	47.5 %	47.5 %	20.0 %	62.1 %	50.0 %
	Specificity	93.7 %	96.3 %	96.3 %	85.7 %	96.3 %	97.6 %	70.0 %
CD56_HBME1_GAL3_CK19	Sensitivity	40.2 %	52.9 %	40.0 %	40.0 %	13.3 %	52.9 %	5.0 %
	Specificity	98.7 %	100 %	100.0 %	92.9 %	100.0 %	100.0 %	90.0 %

*FA* follicular adenoma, *HA* Hurthle cell adenoma, *FTC* follicular thyroid carcinoma, *HCC* Hurthle cell carcinoma, *FVPTC* folicular variant of papillary thyroid carcinoma, non-tumor (hyperplastic and colloid adenomas)

Review of literature was presented within tables (Tables 8, 9, 10, 11 and 12). Sensitivity and specificity for different combinations of entities, taking into account all available cases from reviewed studies, including current study is discussed (Table 13).

**Table 8:** Number of studies, patients and their distributions included in each analysis by the immunohistochemistry technique

	Studies	Patients	TP	FP	FN	TN
CK19	24	4239	1834	436	464	1505
CD56	10	1226	158	418	619	31
HMBE-1	26	4691	1824	311	563	1993
GALECT 3	38	5426	2344	442	458	2182

**Table 9:** Sensitivity and specificity of CK 19 for malignancy, plus total number and percent of cases with positive immunoexpression

Reference	Benign		Malignant		NG		FA		HA		HCA		FTC		PTC		HCC		FVPTC		Sens	Spec	Cut off	TMA	Total
	N	%	N	%	N	%	N	%	N	%	N	%	N	%	N	%	N	%	N	%					
Dunđerović (2015)	79	29 %	122	75 %	30	37 %	27	22 %	12	0 %	10	60 %	15	33 %	87	86 %	20	60 %	40	78 %	75 %	71 %	10 %	yes	201
Barroeta (2006) [14]	53	34 %	37	70 %	4	25 %	3	0 %	18	17 %	3	0 %	7	43 %	11	91 %	4	25 %	4	100 %	70 %	66 %	25 %	yes	90
Zhu X (2010) [15]	58	26 %	180	79 %	17	12 %	21	14 %					11	27 %	155	87 %			7	86 %	79 %	74 %	5 %	no	238
Park YJ (2007) [10]	89	17 %	206	90 %	54	9 %	35	29 %					25	44 %	181	98 %			17	100 %	90 %	83 %	10 %	no	295
Scognamiglio(2006) [16]	49	14 %	78	96 %			49	14 %							78	96 %			29	90 %	96 %	86 %	10 %	yes	127
Nasr MR (2006) [17]	57	68 %	51	100 %	37	75 %	6	83 %	14	43 %					51	100 %			10	100 %	100 %	32 %	10 %	no	108
Prasad ML (2005) [18]	123	11 %	85	66 %	88	15 %	19	10 %	14	0 %	2	0 %	6	50 %	67	72 %	8	50 %	13	79 %	66 %	89 %	10 %	no	208
Casey MB (2003) [19]	30	40 %	30	100 %					30	40 %					30	100 %					100 %	60 %	>0 %	no	60
Erkilic S (2002) [20]	40	28 %	25	100 %	25	20 %	15	40 %							25	100 %					100 %	73 %	NA	no	65
Sahoo S (2001) [21]	20	100 %	15	100 %			20	100 %							15	100 %			10	100 %	100 %	0 %	NA	no	35
Cheung CC (2001) [22]	75	20 %	157	61 %	40	20 %	35	3 %					4	0 %	138	66 %	7	29 %	84	57 %	61 %	80 %	NA	no	232
Barut F (2010) [23]	393	22 %	65	92 %			22	50 %	283	0 %	8	50 %	10	50 %	55	100 %			14	100 %	92 %	78 %	10 %	no	458
Wiseman SM (2008) [24]	100	46 %	105	95 %																	95 %	54 %	NA	yes	205
Liu YY (2008) [25]	100	3 %	77	56 %	64	4 %	12	0 %					13	0 %	53	78 %			11	22 %	56 %	98 %	other	yes	177
Murphy K (2008) [26]	45	47 %	43	65 %	19	89 %	15	27 %					14	43 %	29	76 %			9	22 %	65 %	54 %	>0 %	yes	88
Nakamura N (2006) [27]	32	44 %	130	81 %	5	40 %	27	44 %					21	38 %	94	94 %			45	91 %	81 %	56 %	10 %	yes	162
Rossi ED (2006) [28]	58	3 %	42	86 %			17	2 %	41	2 %					42	86 %			28	82 %	86 %	97 %	NA	no	100
Song Q (2011) [29]	151	26 %	441	96 %	97	26 %	54	24 %							441	96 %					96 %	74 %	10 %	no	592
Beesley MF (2002) [30]	28	25 %	41	83 %	8	25 %	20	25 %					12	41 %	26	100 %					83 %	75 %	>0 %	no	69
de Matos PS (2005) [31]	210	7 %	129	54 %	170	0 %	18	33 %	12	17 %			38	21 %	84	73 %			25	52 %	54 %	93 %	>0 %	no	339
Nechifor-Boilă A (2014) [11]	11	0 %	11	45 %	3	0 %	5	0 %							11	46 %			5	0 %	45 %	100 %	10 %	yes	22
Jang MH (2015) [32]	113	6 %	79	13 %	41	7 %	72	6 %					79	13 %							13 %	94 %	10 %	yes	192
Yassin FE (2015) [33]	18	33 %	24	100 %			8	25 %	4	0 %					24	100 %			6	100 %	100 %	67 %	>0 %	no	42
Choi YL (2005) [34]	9	22 %	125	80 %	9	22 %							30	47 %	67	100 %	6	67 %			90 %	78 %	5 %	no	134
Total	1941		2298		711		500		428		23		285		1764		45		357						4239

*NG/N* nodular goiter/normal, *FA* follicular adenoma, *HA* hyperplastic adenoma, *HCA* Hurthle cell adenoma, *FTC* follicular thyroid carcinoma, *HCC* Hurthle cell carcinoma, *FVPTC* folicular variant of papillary thyroid carcinoma, *Sens* sensitivity, *Spec* specificity, *TMA* tissue microarray, *NA* not available

**Table 10:** Sensitivity and specificity of HBME-1for malignancy, plus total number and percent of cases with positive immunoexpression

Reference	Benign		Malignant		NG		FA		HA		HCA		FTC		PTC		HCC		FVPTC		Sens	Spec			
	N	%	N	%	N	%	N	%	N	%	N	%	N	%	N	%	N	%	N	%			cut off	TMA	Total
Dunđerović (2015)	79	15 %	122	71 %	30	3 %	27	15 %	12	0 %	10	70 %	15	27 %	87	76 %	20	85 %	40	65 %	71 %	85 %	10 %	yes	201
Barroeta (2006) [14]	53	10 %	37	70 %	4	25 %	3	33 %	18	6 %	3	0 %	7	71 %	11	91 %	4	25 %	4	75 %	70 %	90 %	25 %	yes	90
Zhu X (2010) [15]	58	47 %	180	92 %	17	35 %	21	52 %					11	82 %	155	95 %			7	100 %	92 %	54 %	5 %	no	238
Park YJ (2007) [10]	89	32 %	206	91 %	54	20 %	35	49 %					25	88 %	181	92 %			17	94 %	91 %	69 %	10 %	no	295
Scognamiglio (2006) [16]	49	4 %	78	87 %			49	4 %							78	87 %			29	86 %	87 %	96 %	10 %	yes	127
Nasr MR (2006) [17]	57	7 %	51	96 %	37	11 %	6	0 %	14	0 %					51	96 %			10	90 %	96 %	93 %	10 %	no	108
Prasad ML (2005) [18]	123	2 %	92	72 %	88	1 %	19	10 %	14	0 %	2	0 %	6	50 %	67	85 %	8	13 %	13	93 %	72 %	98 %	10 %	no	215
Casey MB (2003) [19]	30	3 %	30	100 %					30	3 %					30	100 %					100 %	97 %	>0 %	no	60
Cheung CC (2001) [22]	75	0 %	157	54 %	40	0 %	35	0 %					4	50 %	138	55 %	7	29 %	84	45 %	54 %	100 %	ukn	no	232
Barut F (2010) [23]	393	5 %	65	97 %			22	50 %	283	1 %	8	25 %	10	90 %	55	98 %			14	100 %	97 %	95 %	10 %	no	458
Wiseman SM (2008) [24]	100	5 %	105	54 %																	54 %	95 %	ukn	yes	205
Liu YY (2008) [25]	100	2 %	77	66 %	64	2 %	12	11 %					13	17 %	53	74 %			11	89 %	66 %	98 %	other	yes	177
Nakamura N (2006) [27]	32	9 %	130	77 %	5	0 %	27	11 %					21	38 %	94	97 %			45	96 %	77 %	91 %	10 %	yes	162
Rossi ED (2006) [28]	58	2 %	42	93 %			17	2 %	41	0 %					42	93 %			28	93 %	93 %	98 %	ukn	no	100
de Matos PS (2005) [31]	210	11 %	129	80 %	170	0 %	18	56 %	12	33 %			38	63 %	84	94 %			25	84 %	80 %	89 %	>0 %	no	339
Nikiforova MN (2003) [36]	23	13 %	33	34 %			23	13 %			13	NA	33	34 %			19	NA			34 %	87 %	10 %	no	56
Mai KT (2002) [37]	93	14 %	96	71 %			45	42 %			48	8 %	29	65 %	55	85 %	12	0 %			71 %	86 %	>0 %	no	189
Liang HS (2009) [38]	48	29 %	71	92 %			48	29 %					26	92 %	45	91 %					92 %	71 %	5 %	yes	119
Ito Y (2005) [39]	253	26 %	175	69 %			155	30 %	98	17 %			138	61 %	37	100 %					69 %	74 %	>0 %	no	428
Mase T (2003) [40]	124	20 %	81	84 %	62	13 %	62	27 %					39	85 %	36	97 %					84 %	80 %	25 %	no	205
Volante M (2004) [41]	50	12 %	102	53 %							50	12 %			32	88 %	70	37 %			53 %	88 %	>0 %	no	152
Nechifor-Boilă A (2014) [11]	11	27 %	11	64 %	3	33 %	5	40 %							11	64 %			5	60 %	64 %	73 %	10 %	yes	22
Jang MH (2015) [32]	113	34 %	79	66 %	41	10 %	72	47 %					79	66 %							66 %	66 %	10 %	yes	192
Guo Z (2015) [42]	52	0 %	85	77 %	20	0 %	32	0 %					28	40 %	57	96 %			29	92 %	77 %	100 %	>0 %	yes	137
Abd-El Raouf (2014) [35]	22	23 %	28	89 %	13	23 %	9	22 %					5	80 %	23	91 %			5	80 %	89 %	77 %	10 %	no	50
Choi YL (2005) [34]	9	33 %	125	90 %	9	33 %							30	100 %	67	97 %	6	67 %			90 %	67 %	5 %	no	134
Total	2304		2387		657		742		522		134		557		1489		146		366		77 %	89 %			4691

*NG/N* nodular goiter/normal, *FA* follicular adenoma, *HA* hyperplastic adenoma, *HCA* Hurthle cell adenoma, *FTC* follicular thyroid carcinoma, *HCC* Hurthle cell carcinoma, *FVPTC* folicular variant of papillary thyroid carcinoma, *Sens* sensitivity, *Spec* specificity, *TMA* tissue microarray, *NA* not available, *ukn* unknown

**Table 11:** Sensitivity and specificity of Galectin 3 for malignancy, plus total number and percent of cases with positive immunoexpression

Reference	Benign		Malignant		NG		FA		HA		HCA		FTC		PTC		HCC		FVPTC						
	N	%	N	%	N	%	N	%	N	%	N	%	N	%	N	%	N	%	N	%	Sens	Spec	Cut off	TMA	Total
Dunđerović (2015)	79	35 %	122	89 %	30	13 %	27	41 %	12	67 %	10	50 %	15	73 %	87	92 %	20	85 %	40	93 %	89 %	65 %	10 %	Yes	201
Barroeta et al. 2006 [14]	53	34 %	37	73 %	4	0 %	3	0 %	18	6 %	3	33 %	7	57 %	11	82 %	4	75 %	4	75 %	73 %	66 %	25 %	Yes	90
Zhu X et al. 2010 [15]	58	34 %	180	86 %	17	24 %	21	14 %					11	64 %	155	92 %				100 %	86 %	66 %	5 %	No	238
Park YJ et al. 2007 [10]	89	5 %	206	95 %	54	6 %	35	3 %					25	64 %	181	99 %			17	94 %	95 %	96 %	10 %	No	295
Scognamiglio (2006) [16]	49	18 %	78	94 %			49	18 %							78	94 %			29	90 %	94 %	82 %	10 %	Yes	127
Prasad ML e (2005) [18]	123	15 %	92	92 %	88	18 %	19	10 %	14	7 %	2	0 %	6	66 %	67	94 %	8	88 %	13	100 %	92 %	85 %	10 %	No	215
Casey MB (2003) [19]	30	60 %	30	100 %					30	60 %					30	100 %					100 %	40 %	0 %	No	60
Barut F (2010) [23]	393	4 %	65	94 %			22	36 %	283	0 %	8	25 %	10	90 %	55	95 %			14	98 %	94 %	96 %	10 %	No	458
Wiseman SM (2008) [24]	100	23 %	105	87 %																	87 %	77 %	ukn	Yes	205
Liu YY (2008) [25]	100	6 %	77	71 %	64	10 %	12	0 %					13	33 %	53	92 %			11	33 %	71 %	94 %	other	Yes	177
Murphy K (2008) [26]	45	42 %	43	65 %	19	42 %	15	33 %					14	21 %	29	86 %			9	55 %	65 %	58 %	>0 %	Yes	88
Nakamura N (2006) [27]	32	25 %	130	88 %	5	0 %	27	30 %					21	48 %	94	96 %			45	98 %	88 %	75 %	10 %	Yes	162
Rossi ED (2006) [28]	58	0 %	42	88 %			17	0 %	41	0 %					42	88 %			28	86 %	88 %	100 %	ukn	No	100
Song Q (2011) [29]	151	51 %	441	97 %	97	52 %	54	48 %							441	97 %					97 %	49 %	10 %	No	592
Beesley MF (2002) [30]	28	18 %	41	85 %	8	38 %	20	10 %					12	100 %	26	85 %					85 %	82 %	>0 %	No	69
de Matos PS (2005) [31]	210	5 %	129	53 %	170	0 %	18	11 %	12	8 %			38	21 %	84	73 %			25	52 %	53 %	95 %	>0 %	No	339
Nikiforova MN (2003) [36]	23	4 %	33	15 %			23	4 %					33	15 %							15 %	96 %	10 %	No	56
Inohara H (2008) [45]	60	28 %	56	89 %	34	24 %	26	35 %					16	63 %	40	100 %					89 %	72 %	>0 %	No	116
Aiad HA (2008) [46]	38	11 %	41	93 %	19	11 %	19	11 %					13	92 %	28	93 %					93 %	90 %	>0 %	No	79
Sapio MR (2007) [47]	62	27 %	106	87 %	51	29 %	11	18 %					32	75 %	74	92 %			19	79 %	87 %	73 %	10 %	No	168
Galusca B (2005) [48]	62	18 %	23	96 %	36	8 %	16	31 %			10	30 %	6	100 %	15	93 %					96 %	82 %	>0 %	No	85
Nucera C (2005) [49]	105	11 %	28	68 %	20	0 %	22	45 %					6	66 %	15	80 %	4	75 %			68 %	90 %	NA	NA	133
Weber KB (2004) [50]	13	31 %	33	79 %			13	31 %					9	44 %	24	92 %					79 %	69 %	5 %	No	46
Oestreicher-Kedem (2004) [70]	25	28 %	29	75 %			19	11 %			6	83 %	11	63 %	18	83 %			18	83 %	75 %	72 %	5 %	No	54
Volante M (2004) [41]	50	12 %	102	95 %							50	12 %			32	97 %	70	94 %			95 %	88 %	>0 %	No	152
Torres-Cabala C (2004) [51]	91	9 %	60	77 %	57	0 %	12	17 %	14	0 %	3	33 %	13	62 %	34	91 %	4	100 %	12	92 %	77 %	91 %	>0 %	No	151
Lavra L (2011) [52]	14	0 %	29	93 %					14	0 %			3	67 %	24	96 %	2	100 %	9	100 %	93 %	100 %	ukn	No	43
Kovacs RB (2003) [53]	55	22 %	36	89 %	25	0 %	19	21 %	3	0 %			10	67 %	20	100 %					89 %	78 %	>0 %	No	91
Jakubiak-Wielganowiczv [54]	42	19 %	42	86 %			42	19 %					17	71 %			25	96 %			86 %	81 %	>0 %	No	84
Giannini R (2003) [55]	20	10 %	45	93 %			20	10 %							45	93 %					93 %	90 %	NA	NA	65
Gaffney RL (2003) [56]	14	21 %	81	85 %			14	21 %					21	67 %	60	92 %					85 %	79 %	other	No	95
Martins L (2002) [57]	60	32 %	32	94 %	29	14 %	31	45 %					20	90 %	12	100 %					94 %	68 %	other	No	92
Coli A (2002) [58]	52	46 %	38	92 %			27	63 %	25	28 %			5	60 %	28	100 %	1	100 %			92 %	54 %	>0 %	No	90
Nascimento MC (2001) [59]	82	2 %	42	71 %	48	0 %	9	11 %			14	7 %	14	79 %	11	82 %	17	59 %			71 %	98 %	>0 %	No	124
Nechifor-Boilă A (2014) [11]	11	0 %	11	46 %	3	0 %	5	0 %							11	46 %			5	0 %	46 %	100 %	10 %	Yes	22
Jang MH (2015) [32]	113	4 %	79	8 %	41	2 %	72	6 %					79	8 %							8 %	96 %	10 %	Yes	192
Abd-El Raouf SM (2014) [35]	22	9 %	28	93 %	13	7 %	9	11 %					5	80 %	23	96 %			5	80 %	93 %	91 %	10 %	No	50
Manivannan P (2012) [60]	12	17 %	10	100 %									3	100 %					7	100 %	100 %	83 %	10 %	No	22
Total	2624		2802		932		748		466		106		488		1947		155		310						5426

*NG/N* nodular goiter/normal, *FA* follicular adenoma, *HA* hyperplastic adenoma, *HCA* Hurthle cell adenoma, *FTC* follicular thyroid carcinoma, *HCC* Hurthle cell carcinoma, *FVPTC* folicular variant of papillary thyroid carcinoma, *Sens* sensitivity, *Spec* specificity, *TMA* tissue microarray, *NA* not available, *ukn* unknown

**Table 12:** Sensitivity and specificity for malignant cases of CD56 (total number and percent of cases with positive immunoexpression)

Reference	Benign		Malignant		NG		FA		HA		HCA		FTC		PTC		HCC		FVPTC						
	N	%	N	%	N	%	N	%	N	%	N	%	N	%	N	%	N	%	N	%	Sens	Spec	Cut off	TMA	Total
Dunđerović (2015)	79	92 %	122	42 %	30	87 %	27	96 %	12	100 %	10	90 %	15	73 %	87	25 %	20	90 %	40	38 %	58 %	92 %	10 %	yes	201
Shahebrahimi K (2013) [64]	39	92 %	39	5 %	32	NA	7	NA							39	5 %			5	NA	95 %	92 %	0 %	no	78
Nechifor-Boilă A (2014) [11]	11	64 %	11	18 %	3	33 %	5	60 %							11	18 %			5	20 %	82 %	64 %	10 %	yes	22
Abd El Atti RM (2012) [65]	44	93 %	29	17 %			12	92 %	32	88 %					29	17 %			16	19 %	83 %	93 %	10 %	no	73
Scarpino S (2007) [61]	107	100 %	66	27 %			26	100 %							61	30 %			21	10 %	73 %	100 %	other	no	173
ElDemellawy D (2009) [62]	100	100 %	75	0 %									2	NA	72	0 %	1	NA	23	0 %	100 %	100 %	10 %	no	175
Satoh F (2001) [68]	11	46 %	39	8 %			11	46 %					10	0 %	14	7 %					92 %	46 %	0 %	no	50
Park WY (2009) [63]	36	92 %	112	38 %	21	91 %	15	93 %					23	83 %	67	8 %					63 %	92 %	10 %	no	148
Nechifor-Boila A (2013) [69]	NA	83 %	204	15 %											204	15 %			90	27 %	85 %	83 %	10 %	yes	204
Mi KS (2011) [66]	22	73 %	80	5 %	12	58 %	5	100 %							80	5 %					95 %	73 %	10 %	no	102
Total	449		777		98		108		44				50		664		21		200						1226

*NG/N* nodular goiter/normal, *FA* follicular adenoma, *HA* hyperplastic adenoma, *HCA* Hurthle cell adenoma, *FTC* follicular thyroid carcinoma, *HCC* Hurthle cell carcinoma, *FVPTC* folicular variant of papillary thyroid carcinoma, *NA* not available, *Sens* sensitivity, *Spec* specificity, *TMA* tissue microarray, *NA* not available

**Table 13:** Sensitivity and specificity (summary of all reviewed studies)

		M/B^a^	PTC/FA	FVPTC/ FA	FVPTC/FTC	FTC/FA	PTC/NT^b^	HCC/HCA
CK19	Sensitivity	80 %	90 %	74 %	74 %	28 %	90 %	51 %
	Specificity	78 %	77 %	77 %	72 %	77 %	82 %	57 %
HBME1	Sensitivity	76 %	88 %	77 %	77 %	64 %	88 %	35 %
	Specificity	87 %	73 %	73 %	36 %	73 %	93 %	57 %
GAL 3	Sensitivity	84 %	93 %	81 %	81 %	50 %	93 %	88 %
	Specificity	83 %	78 %	78 %	50 %	78 %	86 %	77 %
CD56	Sensitivity	80 %	86 %	78 %	78 %	40 %	86 %	10 %
	Specificity	93 %	83 %	83 %	60 %	83 %	54 %	90 %

^a^Malignant vs Benign; ^b^Papillary thyroid carcinoma vs Non Tumor Tissue

## Discussion

This tissue microarray based study is a unique in a methodological way because it gives information of expression of investigated markers on tumour front. It provides answers to questions asked in introductory part of this paper. We provided the readers with extensive review of literature on the subject, with fast insights in methodological approach, cut off values and results of reviewed studies.

All analysed immunomarkers may make a difference between benign lesions/tumours from differentiated thyroid carcinomas. CK19 and HBME-1 can differentiate between papillary carcinoma and follicular carcinoma. Expression of all markers is significantly higher in papillary carcinoma than in follicular adenoma. Galectin-3 could not distinct papillary from follicular carcinoma. CD56 and Galectin-3 could not differentiate between follicular variant of papillary carcinoma and follicular carcinoma. Interestingly, Galectin-3 and CD56 made statistically significant difference between follicular carcinoma and follicular adenoma. The only marker which makes statistically significant difference between adenoma and carcinoma of Hurthle cells was Galectin 3. Best balanced marker in our study, in terms of sensitivity and specificity for malignancy, was HBME-1. The most sensitive marker was Galectin 3, and the most specific marker in our study was CD56. Former mentioned marker, at the same time, had the lowest specificity, and latter had the lowest sensitivity of all. When we combined two, three or four co-expressed markers, we did not reach specificity of 100 % for malignancy, nonetheless values increased compared to single marker expression. On the other hand, the unfavourable result was lowering of sensitivity for malignancy, compared with use of single marker. The best combination of co-expressing markers for identifying malignancy was HBME-1 and Gal-3. Best discriminatory combinations of markers for papillary carcinoma from follicular adenoma, and non-neoplastic lesions were CD56 with Galectin 3, and CK19 with Galectin 3, respectively. For discriminating follicular variant of papillary carcinoma from follicular adenoma or carcinoma, best combinations were CK19 with Galectin 3, and CD56 with HBME1, respectively.

Cytokeratin 19 is the smallest member of cytokeratins family, a heterogeneous group of intermediate filaments. Physiologically, it is expressed in simple and glandular epithelia, basal layer of stratified epithelium, and in hair follicles [12]. Healthy thyroid follicular cells do not produce this protein, and upregulation of CK19 is connected with neoplastic transformation. Strong and diffuse immunoreactivity of CK19 is most often related to papillary thyroid carcinoma [13].

Our result show that CK19 is overexpressed in papillary thyroid carcinoma, diffusely and intensively in most cases. Overexpression of this marker is related not just to PTC, but to malignancy. Discouraging thing, is that serious number of benign cases also expressed CK19, although focally and weakly. Only hyperplastic adenomas were completely negative in our investigation.

We reviewed 23 papers [10, 11, 14–34], and than recalculated average sensitivity and specificity for carcinomas (80 %, 78 % respectively). The results of our study are in concordance with the results from majority of papers we have reviewed. Sensitivity values varied from 13 to 100 % (median_sens_ = 84 %), while specificity values varied from 0 to 100 % (median_spec_ = 75 %) (see Table 9). Control group non tumor tissues, follicular adenoma, follicular carcinoma, and papillary carcinoma had positive expression of this marker which varied from study to study: 4–89 % (median = 20 %); 0–100 % (median = 24 %); 0–50 % (median = 40 %); 46–100 % (median = 95 %), respectively.

Generally, studies showed that CK19 is more expressed in malignant lesions than in benign follicular cells derived thyroid lesions [14, 15]. The expression of CK19 in papillary carcinoma (general) and follicular variant PTC was significantly higher than in follicular thyroid carcinoma (FTC) [15]. Further, it can serve to differentiate papillary thyroid carcinoma from follicular adenoma [31], but can not help in differentiation between follicular adenoma and follicular carcinoma [30, 10]. Also, it does not make difference between follicular carcinoma and normal tissue [26].

In the end, we may make few conclusions: CK19 can help in diagnosis of papillary thyroid carcinoma, but one must take into account the intensity and distribution of marker within the tumour; CK19 intensive immunoreactivity, plus missing criteria for PTC, should alert on possibility of malignancy; due to its relatively low specificity, we recommend the use of marker in combination with others.

HBME1 (Hector Battifora Mesothelial 1), is monoclonal antibody which reacts with uncharacterized antibody in microvilli of mesothelial cells. HBME-1 has been assessed in thyroid with the aim to help in differentiation of benign from malignant lesions.

HBME-1 is more expressed in malignant lesions compared to benign lesions [10, 14, 15, 18, 23, 25, 32, 35]. Benign lesions expressed HBME-1 focally, rather than diffusely. It is more extensively expressed in papillary carcinomas compared to follicular carcinomas and follicular adenomas [18, 25]. Follicular variant of papillary carcinoma has significantly higher expression of this marker than follicular adenoma or follicular carcinoma [25, 31]. The aforementioned studies and their results are in concordance with our study. The results of a few other studies were showing higher expression of HBME-1 in follicular carcinoma than in follicular adenoma. [10, 27, 32, 35], which is not the case with results of our study and some other studies [15, 25, 31]. When we pulled out all the results of expression of HBME-1 in follicular adenomas and follicular carcinomas from reviewed studies, and made comparison of those two groups, we have obtained significant difference in expression between two above mentioned groups (Fisher’s exact test, the two-tailed *P* value is less than 0.0001).

Among 25 reviewed studies [10, 11, 14–19, 22–25, 27, 28, 31, 32, 34–42], the sensitivity and specificity of this marker varied markedly (34–100 % for sensitivity, 54–100 % for specificity), with average sensitivity of 76 % and specificity of 87 % for carcinomas compared to benign lesions (median_sens_ = 77 %; median_spec_ = 89 %). Control non tumor tissues showed positive expression of this marker in the range 0–35 % (median = 10 %) of cases. Follicular adenomas, follicular carcinomas and papillary carcinomas through the studies show positive expression in given ranges: 0–56 % (median = 25 %); 17–100 % (median = 65 %); 55–100 % (median = 92 %), respectively. The increasing trend of expression is noticeable, starting from non-tumour tissues to papillary carcinomas.

Simultaneous immunopositivity for HBME-1 and Galectin 3, and HBME-1 and CK19 in the diagnosis of differentiated thyroid carcinoma have sensitivities of 85,9 %, and 86.4 % respectively, and specificities of 100 % for both combinations. Specificities values increased, but sensitivities values decreased comparing to single markers values [10].

Co-expression of HBME1 and CK19 has a sensitivity of 83 % and specificity of 100 % of diagnosing papillary carcinoma compared to follicular adenoma. Opposite, the HBME1-CK19 negative staining for both markers was highly indicative of follicular adenoma (99 % specificity and 82 % sensitivity) [16].

The combined use of HBME-1 and Gal-3 was able to improve sensitivity up to 99 % and specificity up to 80 % in diagnosis of malignant Hurthle cell tumours compared to Hurthle cell adenomas [41].

Galectin 3 is a structurally unique member (31-kDa) of galectins family. Galectin-3 is capable to make cross links with cell membrane glycoproteins, thus forming new network involved in cellular signaling and receptors endocytosis. Galectin 3 is detected in nucleus, cytoplasm and in extra cellular space. Galectin 3 plays roles in apoptosis regulation, cell motility, and it is involved in thyroid carcinoma progression [43, 44].

Long ago, Xu et al. [44], had been investigating expression of Galectin 1 and Galectin 3 in small series of thyroid tumours, and they had found expression in papillary and follicular carcinomas, but not in adenomas, nodular goiter, nor in normal thyroid tissue. On the grounds of those investigations they deduced that galectins could bu useful in making distinction between benign and malignant thyroid tumours [44].

Expression of Galectin 3 is higher in malignant compared to benign thyroid lesions [10, 14, 15, 18, 23, 25, 35]. Some authors found difference in expression of Galectin 3 between papillary carcinoma, and its follicular variant, compared to follicular carcinoma [10, 25, 31]. Nevertheless, others have not found those differences, including here the results of our study too [15, 35]. Galectin 3 have been found to have higher immunoreactivity in papillary (also follicular variant) carcinoma compared to follicular adenoma [15, 25, 27, 31, 35], which is comparable to our results. Few studies, including the current, found difference in expression of Galectin 3 between follicular carcinoma and follicular adenoma [10, 35]. Galectin 3 was the only marker able to make a difference between Hurthle cell carcinoma and adenoma, which is also confirmed by the study of Volante M et al. [41].

Having made insight in data from reviewed studies [15–19, 23–32, 35, 36, 41, 45–60], once again, we have noticed high variability in sensitivity and specificity for carcinoma of this marker. The values of sensitivity have varied from 8–100 % (median = 88 %), and 40–100 % (median = 82 %) for specificity. The average values were not much different from median values (84 %; 83 %).

The percent of immunopositive cases for control groups (non-tumour), follicular adenomas, follicular carcinomas and papillary carcinomas have varied greatly: 0–52 %(median = 8 %);0–63 %(median = 17 %); 8–100 %(median = 66 %); 46–100 %(median = 93 %), respectively.

Three markers co-expression (HBME1,Gal-3,CK19) had sensitivities of 83 %, 87 %, and 54 % as well as specificities of 100 %, 89 % and 100 %, respectively [14, 16, 27]. Concurrent absence had sensitivity of 38% and specificity of 100 % for benign [14].

In the study of Zhu X et al. [15], more than three immunohistochemical markers were simultaneously positive in 100 % of PTC cases and in 0–30 % of patients with other types of disease.

CD56 is a neural cell adhesion molecule (NCAM), which is expressed normally in thyroid follicular cells. Reduced CD56 expression is correlated with tumour progression of patients with cancer. Its expression is reduced, or totally lost, in cases of papillary carcinoma, follicular carcinoma and anaplastic carcinoma [61–63].

The results of our study showed that malignant tumours had lost or expressed CD56 less than benign thyroid lesions, which is in agreement with previously published data [64–66]. CD56 expression is more reduced in papillary carcinomas in respect to follicular carcinomas and follicular adenomas [62, 63].

On the other side, we had not found significant difference in CD56 expression between follicular variant of papillary carcinoma and follicular carcinoma what is partially supported with results of other study [67]. Intriguingly, we found the difference in expression between follicular carcinoma and follicular adenoma, which is in disagreement with results of other authors [62–64]. If we connect previous information with findings that follicular carcinomas has significantly reduced expression of CD56 relative to benign follicular lesions we could conclude that reduces expression of CD56 is not exclusive property of papillary carcinoma.

Variation among studies in respect to sensitivity and specificity was substantial [11, 61–66, 68, 69]. Sensitivity for carcinoma varied from 58 to 100 % with a median value of 84 % (recalculated average value for all studies is 80 %). Specificity values ranged from 46 to 100 %, with a median value of 92 % (recalculated average value for all studies is 93 %).

The highest sensitivity this marker shows for papillary carcinoma, and the lowest for follicular carcinoma.

Having in mind that we have obtained tissue cores from tumour periphery, our results could be substantiated by the results of other studies [61, 62], which found that within the PTC groups, occasional CD56-positive cells were identified, and in all cases, these cells were located at the tumour/non-tumour interface. Therefore, the low value of sensitivity (58 %) of CD56 in our investigation might be the result of tissue sampling.

Nechifor-Boilă A et al. [11] found that panels consisting of CD56 and/or CK19/Gal-3 (they used Gal-3/CK19 formula because similar results were obtained in the panels containing either CK19 or Gal-3), and CD56 and/or HBME-1 had highest sensitivities (90.9 %) and negative predictive values (87.5 and 83.3, respectively), while the most specific combination of markers was represented by association of HBME-1 with CK19/Gal-3 (72.7 %) in identifying PTC cases. The panel consisting of CD56 and/or HMBE-1 was highly sensitive and specific (100 %, 90 %) in differentiating cases of FVPTC from benign thyroid lesions/tumours. When three-marker panels were evaluated, the best combination for FVPTC cases was HBME-1, CD56 and/or CK19, with a sensitivity reaching 91.1 % [69]. In the studies of Mi KS et al. [66] and W Y Park et al. [63], sensitivities were 93,8 % and 88.1 % and specificities were 90.9 % and 93.8 % respectively, to distinguish PTC from other benign thyroid lesions with the combination of three markers CD56, GAL3, and CK19.

This marked heterogeneity of diagnostic value of investigated markers could stem from few reasons. Primarily, cut off values varied from more than 0 to 25 % of positive cells. Secondarily, some study designs included other tumours beside well differentiated thyroid carcinomas. Further, there is still no consensus about technical points, e.g., HMBE-1 membranous and/or cytoplasmatic staining delivers significant amount of freedom in deciding what is positive. Lastly, some studies employed TMA, others not. At the end, there are results which showed that more than one marker could be co-expressed even in benign lesions, which oblige us to continue the quest of searching more reliable markers. Until then, H&E slides stays golden standard in diagnostics of follicular cells thyroid lesions.

It will not be fair not to mention weaknesses of this study. We shed some light to tumour/normal tissue interface, but we did not have proper representatives of tissue from tumours core. The number of cases, especially Hurthle cell adenomas, and follicular adenomas, was borderline. Some authors also claim that is mistake to include Hurthle cell tumours, but from practical standpoint, because we experienced real dilemmas if tumour is papillary carcinoma (oncocytic variant) or Hurthle cell adenoma for example, we included those tumours also.

## Conclusions

In conclusion we may say that Galectin 3 is most sensitive marker for malignancy, while absence of expression of CD56 is very specific for malignancy. Expected co-expression for combinations of markers in diagnostics of follicular lesions decrease sensitivity and increase specificity for malignancy.

## Abbreviations

FA: Follicular adenoma
FTC: Follicular thyroid carcinoma
FT-UMP: Follicular tumour of uncertain malignant potential
FVPTC: Folicular variant of papillary thyroid carcinoma
HA: Hurthle cell adenoma
HCC: Hurthle cell carcinoma
PTC: Papillary thyroid carcinoma
ROC: Receiver Operating Curve
TMA: Tissue microarray
WDT-UMP: Well differentiated tumours of uncertain malignant potential
